# Infertility Among Active Component Service Women, U.S. Armed Forces, 2019–2023

**Published:** 2025-05-01

## Abstract

This report presents the incidence and prevalence of diagnosed female infertility among active component U.S. service women. During 2019–2023, 8,154 active component women of childbearing potential were diagnosed with incident infertility, resulting in an overall incidence of 77.5 cases per 10,000 person-years (p-yrs). Incidence rates were highest among women in their 30s, non-Hispanic Black individuals, those in health care and pilot or air crew occupations, Army soldiers, and those who were married. From 2019 through 2023, the incidence rate of diagnosed female infertility decreased from 89.2 per 10,000 p-yrs to 69.5 per 10,000 p-yrs despite a concurrent increase in the rate of fertility testing. During the surveillance period, the average annual prevalence of diagnosed female infertility was 1.6%. Of the service women diagnosed with infertility for the first time during the surveillance period, 2,005 (24.6%) delivered live births within 2 years following their incident infertility diagnoses.

For the purposes of public health data collection, the definition of infertility refers to the inability of couples to conceive a pregnancy after 1 year or more of unprotected sex. ^1^ Female infertility is commonly classified into several major etiological categories, including infertility associated with ovulatory dysfunction, tubal disease, uterine and cervical factors, and other or unspecified causes. ^2^ Ovulation disorders are estimated to account for onethird of infertility cases, most often caused by polycystic ovary syndrome (PCOS). ^3 , 4^ Other causes of infertility include hypothalamic-pituitary hormone imbalances, endometriosis, and primary ovarian insufficiency (i.e., premature menopause). ^5^

Advanced maternal age may also contribute to infertility, due to declining egg quality and diminished ovarian reserves. ^6^ Approximately 20% of women in the U.S. now have their first child after age 35 years. ^5^ Data reported by the Department of Defense Birth and Infant Health Registry also indicate a trend toward delayed childbearing in military women. From 2003 to 2014, the percentages of active component women who delivered live births sharply increased among those aged 30-34 (12.5–21.7%) and 35-39 (5.4–8.5%) years. ^7^

Many modifiable lifestyle factors, such as age when starting a family, nutrition, weight, and psychological stress, can have substantial effects on fertility. ^8^ Approximately half of military service women choose to postpone pregnancy or starting a family while in service. ^9^ Occupational and environmental hazards such as radiation, repetitive motions, and injury require more research within military populations for associations with infertility. ^10^ The increasing numbers and durations of wartime deployments have been associated with increasing rates of menstrual disorders and infertility in active component service women. ^11^

**What are the new findings?**

The incidence rate of diagnosed infertility among service women decreased by 22.1% during the surveillance period, coincident with an increase of 74.0% in the rate of fertility testing from 2019 through 2023.

**What is the impact on readiness and force health protection ?**

The incidence rates of diagnosed infertility in the U.S. military reveal that there are sub-groups of active component service members at higher risk. Further assessment of potential risk factors, such as health behaviors, physical as well as mental health conditions, along with occupational exposures, may be warranted. A rapid increase in annual fertility testing rates among active component service women also indicates a need for more comprehensive guidance to infertility service access and use.

*MSMR* has reported the incidence and prevalence of diagnosed female infertility among active component service women in the U.S. Armed Forces since 2000. ^12^ Annual rates of fertility testing have also been assessed since 2019. ^13^ From 2013 to 2018, the incidence of diagnosed female infertility decreased from 85.1 per 10,000 p-yrs to 63.6 per 10,000 p-yrs, despite a concurrent increase in the rate of fertility testing. ^13^

This report continues prior *MSMR* surveillance reporting to provide more recent estimates of the incidence and prevalence of infertility diagnoses, descriptions of specific types of diagnosed infertility, and measures of concurrent rates of fertility testing services among active component service women in the U.S. Armed Forces from 2019 through 2023.

## Methods

The surveillance population consisted of all active component service women of childbearing potential who served in the Army, Navy, Air Force, or Marine Corps at any time from January 1, 2019 through December 31, 2023. Women of childbearing potential were defined as women ages 17-49 years without any history of hysterectomy or permanent sterilization. History of hysterectomy or permanent sterilization was defined by a qualifying diagnostic or procedural code for hysterectomy or permanent sterilization in any position of an inpatient or outpatient record. These diagnostic and procedural codes have been previously described. ^14 , 15^ All data used for these analyses were abstracted from records routinely maintained in the Defense Medical Surveillance System (DMSS) for health surveillance purposes.

An incident case of infertility was defined by at least 2 outpatient medical encounters with an infertility diagnosis (International Classification of Diseases, 9th Revision [ICD-9] code 628.*, International Classification of Diseases, 10th Revision [ICD-10] code N97.*) in the first or second diagnostic position or by an inpatient encounter with an infertility diagnosis in the first diagnostic position. An individual could be counted as a case of infertility only once. The incident date was the date of the first qualifying medical encounter. The type of infertility—either anovulation, tubal origin, uterine origin, other, or unspecified—was assigned from the specific diagnostic codes of the inpatient or outpatient encounter record during military service; however, if an individual had multiple types of infertility, the specific type diagnosed in the earliest incident was utilized **(Table 1)** .

**TABLE 1.** ICD-9 / ICD-10 Codes for Female Infertility

ICD-9	ICD-10	Description
628.0	N97.0	Infertility associated with anovulation
628.2	N97.1	Infertility of tubal origin (block, occlusion, stenosis of fallopian tubes)
628.3	N97.2	Infertility of uterine origin (congenital anomaly of uterus, non-implantation)
628.1, 628.4, 628.8	N97.8	Infertility of other specified origin (pituitary-hypothalamic, cervical or vaginal, age-related, etc.)
628.9	N97.9	Infertility of unspecified origin

Abbreviations: ICD, International Classification of Diseases.

For incidence calculations, person-time denominators were censored at the time of the first hysterectomy or permanent sterilization diagnosis, or when the service member turned 50 years of age, or at the time of the first infertility diagnosis, which-ever occurred first. The incidence rate was calculated per 10,000 person-years (p-yrs).

To be counted as a prevalent case of infertility, the woman of childbearing potential had to 1) be in active component military service during the calendar year of interest, 2) qualify as an incident case of infertility in the year of interest or any year prior (including before 2013), and 3) have an inpatient or outpatient encounter for any infertility type in any diagnostic position during the year of interest. The denominator for prevalence calculations was the total number of women of child-bearing potential in active component service during that year. Prevalence rates were calculated per 10,000 persons.

The burden of medical encounters for infertility was analyzed by calculating the total number of inpatient and outpatient encounters with a primary diagnosis of infertility among all active component service women (including both prevalent and incident cases of infertility). The total numbers of individuals affected and the total number of hospital bed days for infertility were also calculated according to standard *MSMR* burden methodology. ^16^

The rate of fertility testing among all active component women, not just women of childbearing potential, was also measured for the surveillance period. Fertility testing was defined by the presence of an inpatient or outpatient encounter with a diagnosis of fertility testing (ICD-9: V26.21; ICD-10: Z31.41) in any diagnostic position. One test per person per day was counted. The denominator was person-time for all female active component service members during the surveillance period.

Finally, incident infertility cases were followed for up to 2 years to measure sub-sequent live birth deliveries. Live birth deliveries were defined by having a hospitalization with a live birth delivery-related diagnosis code—ICD-9, V27* (excluding V271, V274, V277) and ICD-10, Z37* (excluding Z371, Z374, Z377)—in any diagnostic position.

## Results

### Incidence

**FIGURE 1.**
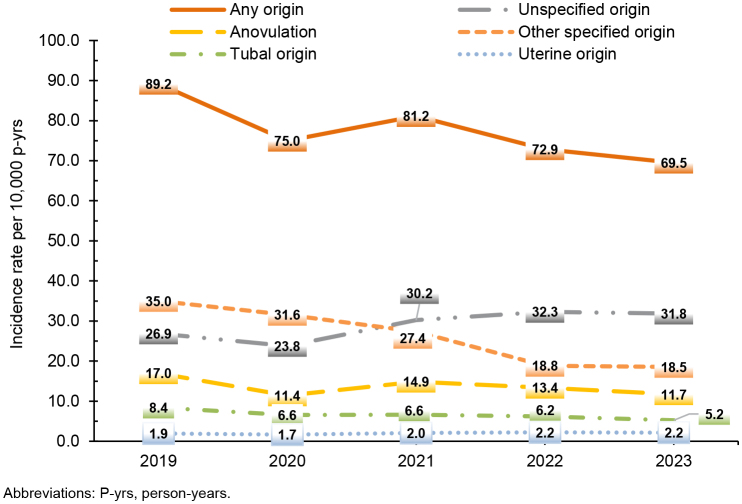
Annual Incidence Rates of Female Infertility Diagnoses, Active Component Service Women of Childbearing Potential, 2019–2023

During the surveillance period, 8,154 active component women of childbearing potential were diagnosed with infertility for the first time, corresponding to a crude overall incidence rate of 77.5 per 10,000 p-yrs **(Table 2)** . Infertility of ‘unspecified’ origin accounted for the most common diagnosis (29.0 per 10,000 p-yrs), followed by ‘other specified’ origin (26.2 per 10,000 p-yrs), and anovulation (13.7 per 10,000 p-yrs). Infertility of tubal origin (6.6 per 10,000 p-yrs) and uterine origin (2.0 per 10,000 p-yrs) represented less common diagnoses for active component women. While the annual incidence of diagnosed infertility (of any origin) decreased by 22.1% during the surveillance period, infertility of unspecified and uterine origin did not follow an overall decline; these 2 infertility types followed the general downward trend in 2019 and 2020, thereafter increasing through 2023 **(Figure 1)** .

**TABLE 2.** Incidence of Infertility by Type, Demographic and Military Characteristics, Active Component Service Women of Childbearing Potential, U.S. Armed Forces, 2019–2023

	No.	Rate ^a^
Total	8,154	77.5
Type of infertility		
Anovulation	1,437	13.7
Tubal origin ^b^	694	6.6
Uterine origin ^c^	211	2.0
Other specified origin	2,761	26.2
Unspecified origin	3,051	29.0
Age, y		
<20	61	6.9
20–24	1,533	40.8
25–29	2,276	83.2
30–34	2,347	143.0
35–39	1,535	159.2
40–44	382	96.7
45–49	20	14.7
Race and ethnicity		
White, non-Hispanic	3,314	75.5
Black, non-Hispanic	2,162	89.3
Hispanic	1,499	68.0
Other / unknown	1,179	78.3
Service branch		
Army	2,959	92.0
Navy	2,365	75.1
Air Force	2,296	74.6
Marine Corps	354	45.3
Coast Guard	169	60.9
Space Force	11	68.5
Rank		
Junior enlisted (E1–E4)	2,492	50.1
Senior enlisted (E5–E9)	3,311	92.3
Junior officer (O1–O3, W01–W03)	1,554	106.4
Senior officer (O4–O10, W04–W05)	797	160.0
Military occupation		
Combat-specific ^d^	200	60.5
Motor transport	221	62.1
Pilot / air crew	167	96.6
Repair / engineering	1,333	63.4
Communications / intelligence	2,645	81.2
Health care	2,016	113.4
Other / unknown	1,572	62.4
Marital status		
Married	5,973	136.0
Unmarried	1,456	28.2
Other	725	74.8

Abbreviations: No., number; y, years.

a Rates per 10,000 person-years.

b Block, occlusion, or stenosis of fallopian tubes.

c Structural abnormality of uterus or non-implantation; includes fibroids.

d Includes infantry / artillery / combat engineering / armor.

**FIGURE 2.**
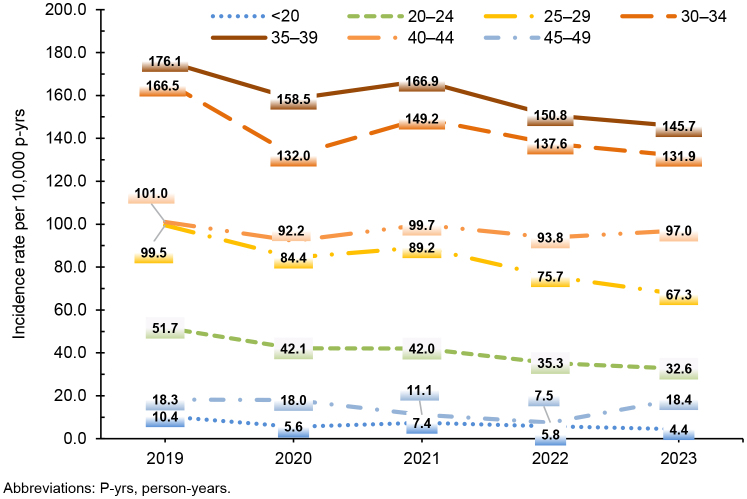
Annual Incidence Rates of Female Infertility Diagnoses by Age Group, Active Component Service Women of Childbearing Potential, 2019–2023

**FIGURE 3.**
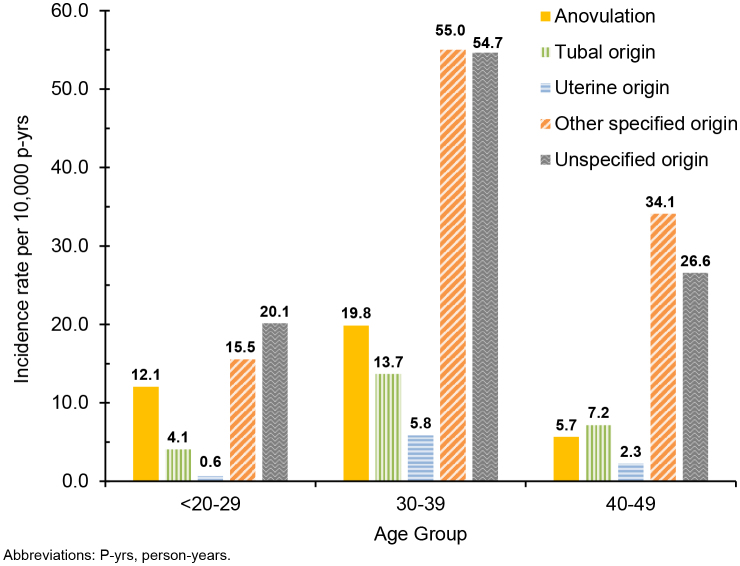
Incidence Rates of Female Infertility Diagnoses by Type and Age Group, Active Component Service Women of Childbearing Potential, 2019–2023

Overall rates of incident infertility diagnoses increased with age, peaking for women ages 35-39 years (159.2 per 10,000 p-yrs) **(Table 2)** . While incident infertility diagnoses for women under age 40 years followed a general decline for the overall surveillance period, rates of incident infertility for those aged 40-44 years remained relatively stable. Among women ages 45-49 years, the incident infertility rate declined to 7.5 per 10,000 p-yrs in 2022, thereafter increasing to 18.4 per 10,000 p-yrs in 2024 **(Figure 2)** . For this oldest age group (40-49 years), infertility due to ‘other specified’ origin accounted for the highest rate of diagnosis (34.1 per 10,000 p-yrs), whereas ‘other specified’ and unspecified origins accounted for approximately equal rates (55.0 and 54.7 per 10,000 p-yrs, respectively) for women aged 30-39 years, and unspecified infertility accounted for the highest rate of diagnosis (20.1 per 10,000 p-yrs) in women 20-29 years of age **(Figure 3)** .

**FIGURE 4.**
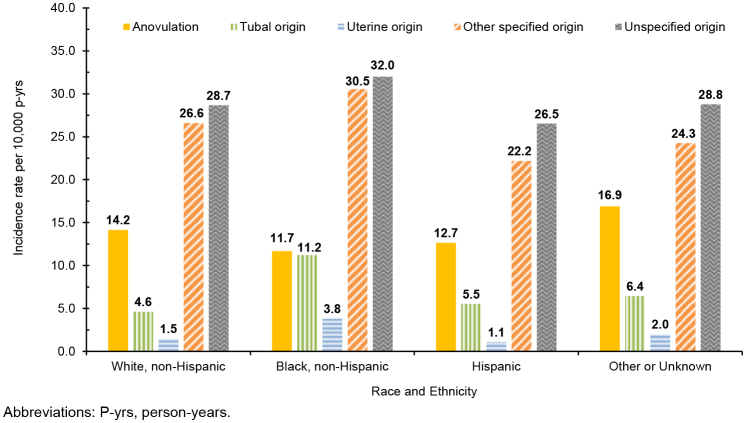
Incidence of Infertility by Type and Race and Ethnicity, Active Component Service Women of Childbearing Potential, U.S. Armed Forces, 2019–2023

Overall incidence rates of infertility diagnoses of any type were highest among non-Hispanic Black service women (89.3 per 10,000 p-yrs) compared to women in other race and ethnicity groups **(Table 2)** ; this finding is consistent for each type of infertility diagnosis, with the exception of anovulation **(Figure 4)** . Active component service women of other or unknown racial and ethnic groups accounted for the highest rates of infertility due to anovulation (16.9 per 10,000 p-yrs), followed by non-Hispanic White (14.2 per 10,000 p-yrs) and Hispanic (12.7 per 10,000 p-yrs) service women.

Overall rates of incident infertility diagnoses were highest among service women in the Army (92.0 per 10,000 p-yrs) and lowest in the Marine Corps (45.3 per 10,000 p-yrs), although it should be noted that these findings present rates that were unadjusted for age **(Table 2)** . Senior enlisted women had higher incidence rates than junior enlisted personnel, and senior officers had higher rates than junior officers. Compared to other occupations, service women in health care occupations had the highest incidence of diagnosed infertility (113.4 per 10,000 p-yrs), and were followed by pilots and air crew (96.6 per 10,000 p-yrs). The rate of incident infertility diagnoses among married service women was nearly 5 times the rate of unmarried service women.

### Prevalence

**FIGURE 5.**
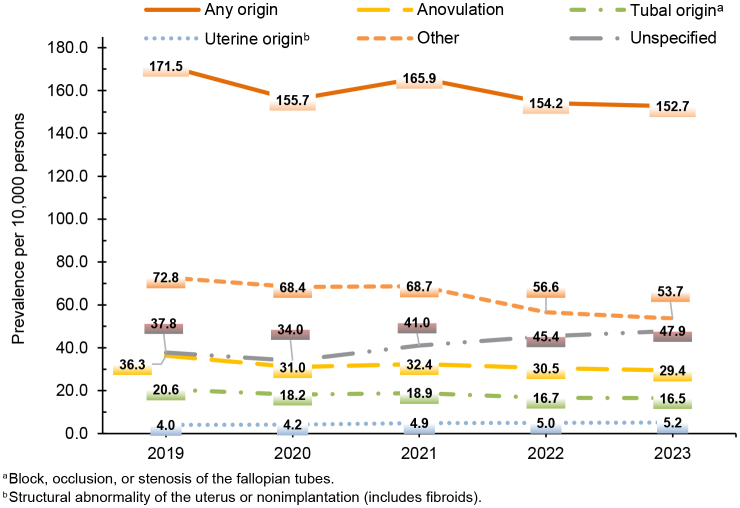
Prevalence of Infertility by Type, Active Component Service Women of Childbearing Potential, U.S. Armed Forces, 2019–2023

In 2023, the prevalence of diagnosed female infertility of any type was 152.7 per 10,000 persons, translating to 1.5% of the female active component population that year. This figure decreased by approximately 11% during the surveillance period, down from 171.5 per 10,000 persons (or 1.7%) in 2019. Two types of infertility increased during the surveillance period: Prevalence of infertility of uterine origin increased by 30.0% (from 4.0 to 5.2 per 10,000 persons) and infertility of unspecified origin rose by 26.8% (from 37.8 to 47.9 per 10,000 persons) from 2019 through 2023 **(Figure 5)** .

### Burden

**FIGURE 6.**
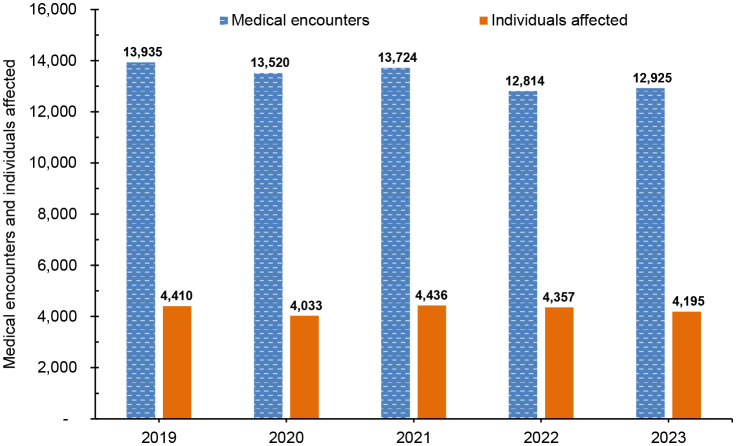
Numbers of Medical Encounters for Infertility and Numbers of Individuals Affected, Active Component Service Women, 2019–2023

There were 66,918 total medical encounters and 31 hospital bed days recorded for female infertility during the surveillance period (data not shown). Annual numbers of medical encounters during which infertility was reported as a primary (first-listed) diagnoses and numbers of individuals affected by infertility remained relatively stable during the period, declining from 13,935 medical encounters in 2019 to 12,925 medical encounters in 2023 **(Figure 6)** .

### Fertility testing

**FIGURE 7.**
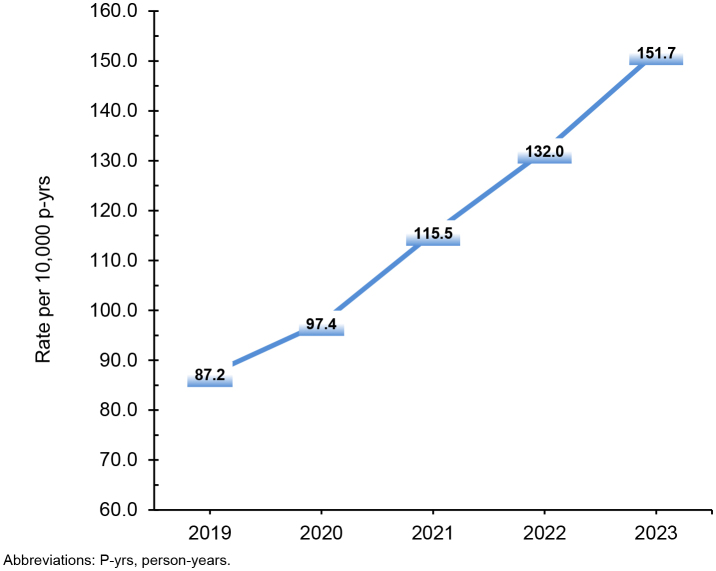
Annual Rates of Fertility Testing, Active Component Service Women of Childbearing Potential, U.S. Armed Forces, 2019–2023

During the surveillance period, annual rates for female fertility testing increased 74.0%, from 87.2 per 10,000 p-yrs in 2019 to 151.7 per 10,000 p-yrs in 2023 **(Figure 7)** .

### Live births after infertility diagnosis

Of the 8,154 service women diagnosed with infertility for the first time during the surveillance period, 666 (8.2%) were hospitalized for a live birth within 1 year following incident their infertility diagnoses (data not shown). In total, 2,005 (24.6%) women were hospitalized for a live birth within 2 years after an incident infertility diagnosis.

## Discussion

The crude overall incidence rate of diagnosed infertility among active component service women during the 2019–2023 surveillance period (77.5 per 10,000 p-yrs) remained slightly below the 2013–2018 incidence rate (79.3 per 10,000 p-yrs) previously reported, despite increased fertility testing. ^13^ Over 70% of incident infertility cases were diagnosed as ‘other specified’ or unspecified origin, limiting descriptions of the types of causes of infertility. While annual incidence rates of diagnosed infertility (of any origin) decreased by 22.1% during the surveillance period, the rate of unspecified infertility increased by nearly 34% from 2020 through 2023. This increasing trend in unspecified diagnoses, coupled with a sustained proportion of cases diagnosed as ‘other’ origin, may warrant further study to better elucidate the specific types of causes of infertility; however, current ICD-10-CM coding does not provide a greater level of detail beyond the unspecified and ‘other’ diagnoses.

The demographic results reported herein are broadly similar to prior surveillance reports focused on active component service women, with the highest rates of female infertility diagnosed among Army soldiers, women of non-Hispanic Black race or ethnicity, and individuals aged 30-39 years. Between 2000 and 2012, active component service women ages 30-34 years accounted for the highest rates of diagnosed infertility, but the highest rates shifted to women ages 35-39 years from 2013 to 2018. This finding has persisted through this surveillance period, 2019–2023, in which women ages 35-39 years accounted for the highest rates of diagnosed infertility, followed closely by women aged 30-34 years. The comparison of age-stratified rates for infertility of uterine origin are also notable for women in their 30s. While the overall incidence rate of women diagnosed with infertility of uterine origin remained minimal during the surveillance period, the age-stratified rate (5.8 per 10,000 p-yrs) for women aged 30-39 years from 2019 to 2023 is elevated beyond the comparable age-specific rate reported for 2013–2018, at 2.9 per 10,000 p-yrs. ^13^

Notably, fertility testing for active component service women increased by 74.0%, exceeding the increasing trend (30.0%) described in the prior surveillance period. ^13^ The current report also approximates that one-quarter (24.6%) of women had live births within 2 years following their incident infertility diagnoses, increasing from one-fifth (20.7%), previously reported for 2013–2018. ^13^

Over the last 3 decades, development of new medications, testing, and treatment strategies for infertile women have increased at a rapid pace. ^17^ Women in active military service may receive diagnostic services to identify physical causes of infertility and some medically necessary treatments (e.g., hormonal therapy, corrective surgery, antibiotics). TRICARE does not currently cover assisted reproductive technology services (ART), except for service-related infertility. ^18^ ART services are available on a ‘first-come, first-serve’ basis at greatly reduced cost, offered at 8 military hospitals with obstetrical / gynecological reproductive endocrinology and infertility graduate medical education programs. ^18 , 19^ Access to these infertility services may vary, depending on a range of factors such as current duty station location, career stage, cost of services, command climate, and current policy. ^20^ As testing services become more commonly used, analyses related to the use, safety, efficacy and quality of infertility treatments may be warranted, based on guidelines from the Centers for Disease Control and Prevention's National Public Health Action Plan for the Detection, Prevention and Management of Infertility. ^10^

The results presented in this report should be interpreted as estimates defined from administrative diagnostic codes, which are methodologically different from studies that use self-reported survey tools. Furthermore, administrative diagnostic codes may underestimate the true incidence and prevalence of infertility. The prevalence estimates from this report (1.5–1.7%) remain far below self-reported data from the Department of Defense Women's Reproductive Health Survey (15.2%), ^9^ due to inherent methodological differences in comparing survey data with diagnostic codes from electronic health records.

Additional limitations may be present in this report. The percentage of women who gave birth following incident infertility diagnoses is likely underestimated, as women who gave birth after leaving military service are not captured. Furthermore, this analysis did not explicitly capture recurrent pregnancy loss (ICD-9: 629.81, 646.3*; ICD-10: N96, O26.2*), which could be considered a type of infertility. Some individuals diagnosed with recurrent pregnancy loss may have received a diagnosis of unspecified infertility, however, and would have been included in this analysis.

Despite these limitations, this report provides an update on the incidence and prevalence of diagnosed infertility among active component U.S. service women. The standardized measurement of diagnosed infertility provides a basis for reviewing trends and comparing rates by socio-demographic variables, in addition to further assessing suggested risk factors, ^1^ such as health behaviors (e.g., alcohol or tobacco use), physical and mental health conditions (sexually transmitted infections, obesity, depression, cancer), and occupational exposures. Furthermore, the rapidly increasing rates of fertility testing among active component service women indicates need for further studies to more comprehensively describe infertility service access and use.
